# Perceived discrimination in middle-aged and older adults: Comparison between England and the United States

**DOI:** 10.3389/fpubh.2022.975776

**Published:** 2022-11-10

**Authors:** Aliya Amirova, Katharine A. Rimes, Ruth A. Hackett

**Affiliations:** Department of Psychology, Institute of Psychiatry, Psychology and Neuroscience, King's College London, London, United Kingdom

**Keywords:** cohort analysis, cross-cultural study, diversity in aging, discrimination, aging

## Abstract

**Objectives:**

This study examined differences in perceived discrimination across multiple characteristics in England and the United States (US), in middle- and older-aged adults.

**Methods:**

Using data from the English Longitudinal Study of Aging (*N* = 8,671) and the US-based Health and Retirement Study (*N* = 7,927), we assessed cross-national differences in perceived discrimination attributed to disability, financial status, sex, race, sexual orientation, and weight. We also compared how perceived discrimination varied with socioeconomic position (SEP) based on wealth.

**Results:**

Perceived discrimination due to financial status was more common in England (6.65%) than in the US (2.14%) adjusting for age, sex, and wealth [Odds Ratio (OR) = 1.09, 95% CI (1.07; 1.10)]. This affected people of low but not high SEP. Sexual orientation discrimination was more common in England [0.72 vs. 0.15%, OR = 4.61, 95% CI (2.48; 8.57)]. Sex-based perceived discrimination was more prevalent in the US (12.42%) than England (9.07%) adjusting for age and wealth [OR = 0.87, 95% CI (0.86; 0.89)]. Cross-national differences in sex discrimination did not vary with SEP. Racism was the most common type of perceived discrimination reported in both samples (England: 17.84%, US: 19.80%), with no significant cross-national differences after adjustment for sex.

**Discussion:**

Perceived discrimination attributed to financial status and sexual orientation were more prevalent in England, while more women perceived sex discrimination in the US. This study suggests that country-specific and socioeconomic factors affect the prevalence of perceived discrimination. This may be relevant when targeting interventions aimed at reducing perceived discrimination.

## Introduction

Globally, populations are aging. By 2050, it is estimated that people aged 65 and over will make up 24 and 21.4% of the population in the United Kingdom (UK) and United States (US), respectively (1, 2). Health and wellbeing at an older age is a policy priority (3). Perceived discrimination is increasingly recognized as a risk factor compromising healthy aging.

Discrimination is the prejudiced and unfair treatment of individuals based on demographic or ascribed characteristics (4) including disability, race, sex, and socioeconomic background (4). Perceived discrimination is associated with poorer mental and physical health (5–7). This is supported by population-based studies of middle-aged and older adults such as the English Longitudinal Study of Aging (ELSA) and the US-based Health and Retirement Study (HRS). ELSA findings suggest that perceived age discrimination is associated with an increased likelihood of chronic illness (8). Other ELSA studies have linked perceived weight (9) and sexual orientation discrimination with depression and lower quality of life (10). In adults with health conditions such as visual impairment (11) and pain (12), perceived discrimination has been shown to negatively impact wellbeing. Analyses of the HRS sample indicate that perceived discrimination due to stable characteristics (e.g., race) is associated with loneliness, while perceived discrimination due to characteristics that can change over time (e.g., disability and weight) is associated with the onset of chronic conditions, lower self-rated health and life satisfaction (13).

Studies have investigated perceived discrimination in the ELSA and HRS cohorts separately. However, cross-national comparisons can offer additional insights. The sociocultural and historical contexts of England and the US differ and thus, may influence discrimination experiences. For example, the make-up of ethnic minority groups in England differs from the US, with those of South Asian backgrounds forming the largest minority group in England (14). While those of Hispanic/Latinx ethnicity represent the largest minority group in the US (15). Additionally, in terms of wealth distribution, England has a history of a hierarchically organized society (16), while the US is perceived as more economically egalitarian (17). Therefore, understanding differences in the prevalence of various types of discrimination may elucidate areas for interventions.

Previous work such as the Eurobarometer survey, including participants from 28 European countries, suggests that perceived discrimination due to ethnicity (64%), sexual orientation (58%), disability (50%), and gender (37%) is perceived to be common with some variability between countries (18). However, the extent of perceived discrimination in middle-aged and older adults is not well documented in such surveys (19, 20), despite evidence that social exclusion is common at these life stages and is linked to poor health (21). Additionally, cross-national comparison of the context, type and rates of perceived discrimination is limited by a lack of comparative measures in existing surveys. Perceived discrimination measures in ELSA and HRS have been harmonized, facilitating cross-national comparison.

To our knowledge, only one study to date has assessed cross-national differences in perceived discrimination between England and the US using cohort data from middle-aged and older adults. This study focused on perceived age discrimination in the ELSA and HRS cohorts and found that more adults in England than in the US reported age-related discrimination (22). Building on this evidence, we aim to assess cross-national differences in perceived discrimination attributed to other characteristics such as disability, financial status, sex, sexual orientation, race and weight in ELSA and HRS. Middle-aged and older adults have heterogeneous characteristics such as race, weight, and financial status (23), so it is important to understand the prevalence of discrimination attributed to these characteristics.

Experiences of discrimination vary depending on socioeconomic position (SEP). Perceived age discrimination was associated with lower wealth in ELSA and HRS samples (22). While, inequalities in education and wealth are well documented in middle and older age (24). Therefore, we aimed to assess cross-national differences in the wealth gradient for perceived discrimination in middle-aged and older adults.

## Materials and methods

### Data source and study design

In a cross-sectional analysis we used data from two nationally representative studies of aging: ELSA in England and the HRS in the US. These studies were designed to be comparable and closely matched in sampling and questionnaire content. Harmonized data files from wave 5 (2010) of ELSA and wave 7 of HRS (2010) were obtained from the Gateway to Global Aging (g2aging.org). Analyses were constrained to 2010 as perceived discrimination was assessed in ELSA at this time point only.

### Study population

**Figure 1:**
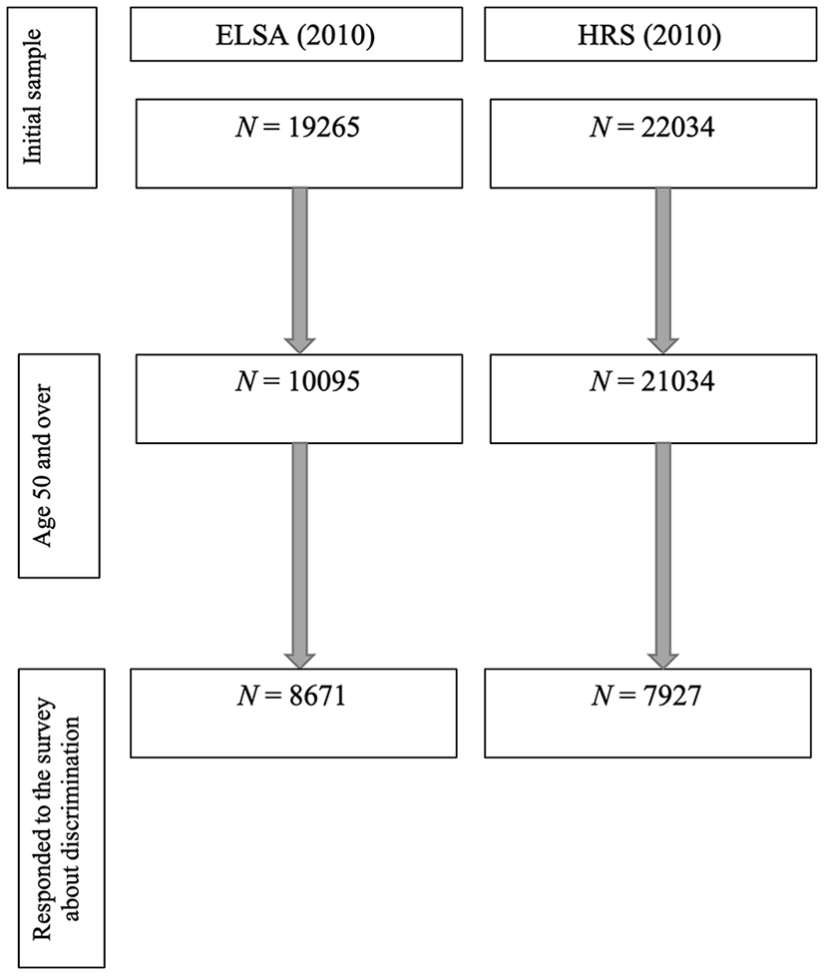
Study flow chart.

ELSA and HRS cohorts have been described in detail elsewhere (25, 26). Analyses were restricted to participants aged ≥ 50 years who provided perceived discrimination data, resulting in analytic samples of *N* = 8,671 (ELSA) and *N* = 7,927 (HRS), Figure 1.

### Ethical approval

ELSA was approved by the London Multicentre Research and Ethics Committee (MREC/01/02/91). Approval for HRS was obtained from the University of Michigan Institutional Review Board (https://hrs.isr.umich.edu/publications/biblio/9048).

### Measures

Perceived discrimination was assessed in both cohorts using the same items based on the frequency of discrimination in five situations: “*In your day-to-day life, how often have any of the following things happened to you (1) you are treated with less respect or courtesy; (2) you receive poorer service than other people in restaurants and stores (ELSA: shops); (3) people act as if they think you are not clever; (4) you are threatened or harassed; (5) you receive poorer service or treatment than other people from doctors or hospitals*” (almost every day/at least once a week/a few times a month/a few times a year/less than once a year/never). As previously reported (27), the data were skewed, with most individuals reporting “never” experiencing discrimination, we created a binary variable to indicate whether participants had experienced discrimination in the past year (a few times or more a year vs. less than once a year or never), except for the fifth item which was dichotomized to indicate whether respondents had ever experienced discrimination from doctors or hospitals (never vs. all other options) as most participants reported “never” experiencing discrimination in this setting. A follow-up question asked participants to attribute the discriminatory experience to one or more reasons: physical disability, financial status, race, sex, sexual orientation, weight, and age. This measure has good validity for the assessment of discrimination (28, 29) and has been widely used in research investigating associations between discrimination and health in ELSA and HRS (5–8, 11, 12, 30).

### Covariates

Age was modeled as a categorical variable (52–59; 60–69; 70–79; 80+ years old). Sex (female/male) was modeled in binary. Wealth (excluding pension wealth) is the most relevant indicator of SEP in these cohorts (25, 26). This measure is based on detailed assessments of socioeconomic resources (e.g., financial wealth, including housing costs, assets, earnings, debts; and physical wealth) (i.e., land and jewelery). We modeled wealth as a continuous and categorical variable based on a cohort-specific median split (below median = low SEP, above the median = high SEP).

### Statistical analysis

Analyses were carried out in R. We first assessed whether the HRS and ELSA samples differed in age, sex, and wealth (our key covariates) using a series of *t*-tests for normally distributed continuous variables (age, wealth) and χ^2^ (chi-square) tests for binary variables (sex and SEP level).

We assessed unadjusted cross-national differences in the prevalence of perceived discrimination attributed to disability, financial status, sex, race, sexual orientation, and weight using multiple logistic regression models and χ^2^-tests. Analyses of perceived disability discrimination were restricted to those living with a longstanding physical limitation (defined as an impairment in basic or instrumental activities of daily living or impaired mobility). Perceived race discrimination analyses were restricted to ethnic minority participants. Perceived sex discrimination analyses were limited to female participants. perceived weight discrimination analyses were restricted to obese participants (Body Mass Index: BMI > 30 kg/m^2^). We choose to restrict all analyses to improve the precision of the estimates so that we compare the prevalence of discrimination in non-dominant groups (e.g., women) who do and do not perceive discrimination.

Sexual orientation was assessed using item: “*Which statement best describes your sexual desires over your lifetime? Please include being interested in sex, fantasizing about sex or wanting to have sex*”: (1) entirely for women, (2) mostly for women, but some desires for men, (3) equally for women and men, (4) mostly for men, but some desires for women, (5) entirely for men, and (6) no sexual desires in lifetime. We categorized participants with reported desires entirely for the opposite sex as heterosexual, entirely for the same sex as gay and those reporting desires equally for both sexes, mostly for the same sex, or some desires for opposite sex as bisexual. The number of lesbian, gay, or bisexual (LGB) individuals was small in both ELSA (*n* = 139) and HRS cohorts (*n* = 9). Therefore, sexual orientation discrimination analysis was not restricted to LGB individuals. As in previous work (22), data were unweighted as we combined two subsamples of HRS and ELSA respondents, which had different weights.

We then conducted logistic regression analyses adjusting for covariates that were significantly associated with each discrimination type as in previous work (22). We implemented generalized linear models (binomial) with perceived discrimination as the independent variable and country (US/England) as the dependent variable. Likelihood ratio tests for two nested models were conducted to assess differences in perceived discrimination between the countries. We also assessed these relationships looking at each of the five discriminatory situations separately.

We performed secondary analyses to compare the role of SEP in the prevalence of perceived discrimination in England and the US. Firstly, we fitted multiple binomial regression models to compare the moderating effect of continuous wealth on each type of perceived discrimination across the two countries. We also re-ran logistic regression analyses stratified by SEP (low/high), removing adjustment for this variable. Secondly, removing the stratification by SEP, we assessed the interactive effects of SEP and country on the prevalence of perceived discrimination attributed to each characteristic separately. A log-likelihood ratio test was used to test for interaction. As in previous work (20), data were unweighted as we combined two subsamples of HRS and ELSA respondents, which had different weights.

### Sensitivity analysis

We carried out the cross-national comparison stratified by SEP operationalized using education level. Education was included as binary variable (no higher degree and higher degree), describing the highest educational qualification attained. No higher degree included no formal education, GCSE, O-Level A-Levels, or equivalent in ELSA; and no formal education, education below high school or completed high school in HRS. Higher degree included university degree or higher in ELSA and college to post-college in HRS. We also included education as a covariate in addition to age, sex, and wealth.

The analysis concerned within-country comparisons (low SEP vs. high SEP and association between wealth and perceived discrimination) was also weighted to test the impact of adjusting for selection bias. We performed weighted logistic regression to account for selection bias in HRS and ELSA separately when assessing the association between wealth and discrimination.

## Results

### Participant characteristics

This study included 8,671 individuals from ELSA [*M* = 66.57 (SD = 9.03) years old; 4816 (55.54%) female] and 7,927 individuals from HRS [*M* = 67.28 (SD = 10.80) years old; 4,583 (57.82%) female] who responded to the perceived discrimination survey (Table 1).

**Table 1:** Participant characteristics.

	**England** **(*N* = 8,671)**	**United States** **(*N* = 7,927)**
**Age (mean**, ***SD*****)**	66.57 (SD = 9.03)	67.28 (SD = 10.80)
**Living with a physical limitation (** ***n*** **, %)**	2,890 (33.33%)	386 (4.87%)
**Women (** ***n*** **, %)**	4,816 (55.54%)	4,583 (57.82%)
**Ethnic minority groups**
Ethnic minorities (total *n*, %)	213 (2.46%)	717 (9.05%)
Black	33 (15.45%)	414 (57.75%)
Asian	49 (23%)	-
Hispanic	-	303 (42.25%)
Mixed	7 (3.29%)	-
Other/did not state	124 (58.2%)	-
**Low SEP**	7,581 (87%)	7,068 (89%)
**Weight (BMI, kg/m** ^**2**^ **)**
BMI (mean, SD)	28.16 (SD = 5)	35.37 (SD = 18.14)
BMI > 30 kg/m^2^ (*n*, %)	1,524 (24.46%)	4,874 (61.58%)

Percentages are valid percent.

BMI, Body Mass Index; SEP, Socioeconomic Position; SD, Standard Deviation.

We observed significant differences in age, sex and SEP between ELSA and HRS samples. Participants in HRS were on average older [*t*_(1)_ = 4.57, *p* < 0.001], more likely to be female (χ^2^ = 8.62, *p* < 0.01), and less wealthy [*t*_(1)_ = −9.26, *P* < 0.001] than those in ELSA. There were no significant differences in number of individuals in low SEP between HRS (*n* = 7,068, 89%) and ELSA (*n* = 7,581, 87%) cohorts, χ^2^ = 0.61, *p* = 0.44.

### Cross-national differences in perceived discrimination

In unadjusted analysis (Table 2), perceived discrimination attributed to disability [OR = 3.05, 95% CI (1.42; 6.55), *p* < 0.01], financial status [OR = 3.31, 95%CI (2.75; 4.00), *p* < 0.001], sexual orientation [OR = 4.61, 95%CI (2.48; 8.57), *p* < 0.001], and weight [OR = 1.41, 95% CI (1.14; 1.75), *p* < 0.01] was more frequently reported in England than the US. Race- [OR = 0.62, 95%CI (0.42; 0.93), *p* < 0.05] and sex-based discrimination [OR = 0.35, 95%CI (0.30; 0.40), *p* < 0.001] were more prevalent in the US than in England.

**Table 2:** Prevalence and cross-national differences in perceived discrimination attributed to disability, financial status, sex, race, sexual orientation, and weight.

**Attribution for discrimination**	**Total** ***n***		**Perceived discrimination**, ***n*** **(%)**				**Unadjusted cross-national difference**					**Adjusted cross-national difference**				
	**England**	**US**	**England**		**US**		**OR^a^**	**95% CI**		**χ^2^**	***p*-value**	**OR**	**95% CI**		**χ^2^**	***p*-value**
Disability (physical limitation)	2,890	386	264	9.13	7	1.81	3.05	1.42	6.55	8.29	**0.0040**	1.06	1.02	1.11	0.75	0.3878
Financial status (low SEP)	7,581	7,068	504	6.65	151	2.14	3.31	2.75	4.00	171.60	**0.0000**	1.10	1.09	1.12	17.10	**0.0000**
Sex (female)	4,816	4,583	437	9.07	569	12.42	0.35	0.30	0.40	243.69	**0.0000**	0.87	0.86	0.89	29.41	**0.0000**
Race (ethnic minority)	213	717	38	17.84	142	19.80	0.62	0.42	0.93	4.86	**0.0275**	0.94	0.86	1.03	0.35	0.5518
Sexual orientation*	8,671*	7,927*	62	0.72	12	0.15	4.61	2.48	8.57	27.00	**0.0000**	NA	NA	NA	NA	NA
Weight (BMI > 30)	1,947	5,192	178	9.14	199	3.83	1.41	1.14	1.75	10.07	**0.0015**	1.03	1.02	1.05	1.14	0.2852

^a^ORs > 1 indicate higher prevalence in England and ORs < 1 indicate the higher prevalence in the US. Values in bold meet the 0.05 *p*-value threshold.

BMI, Body Mass Index; CI, Confidence Interval; OR, Odds Ratio; SEP, socioeconomic status.

OR was adjusted for the following covariates: Disability: age, sex and wealth; Financial status: age and sex; Sex discrimination (female): age and wealth; Sexual orientation: no significant covariates; Race: age, sex and wealth; Weight: age, sex and wealth.

^*^Unrestricted analysis due to small n of lesbian, gay, bisexual (LGB) individuals included in the studies, ELSA [n =139, 10 (7.2%) of whom perceived sexual orientation discrimination] and HRS [n = 9, one (11.1%) of whom perceived sexual orientation discrimination]. Cross-national differences in ageism were previously assessed by Rippon et al. We include the replication of the results in Supplementary Tables 1, 2. The results of the analysis restricted to those with BMI between 25 and 30 kg/m^2^ are included in Supplementary Table 3.

In adjusted analyses, significant cross-national differences in financial status and sex-based perceived discrimination remained. Specifically, perceived financial status discrimination [OR = 1.10, 95%CI (1.09; 1.12), *p* < 0.001] was more common in England than in the US (6.65 vs. 2.14%), adjusting for age, sex, and wealth. Sex discrimination was more prevalent in the US than in England [12.42 vs. 9.07%; OR = 0.87, 95%CI (0.86; 0.89), *p* < 0.001] adjusting for age and wealth).

We also compared perceived discrimination in five discriminatory situations separately (Supplementary Table 4). In unadjusted analyses in England, being treated with less respect was the most prevalent type of discriminatory experience regardless of the attributed cause. In adjusted analyses, being treated with less respect was more often attributed to financial- [OR = 1.45, 95%CI (1.30; 1.63), *p* < 0.01], sex- [OR = 1.87, 95% CI (1.78; 1.96), *p* < 0.001], race- [OR = 1.57 95% CI (1.34; 1.84), *p* < 0.001], and weight discrimination [OR = 1.74, 95% CI (1.54; 1.97), *p* < 0.001], in England than in the US.

In the US, individuals perceived disability (86%) and sexual orientation (75%) discrimination most often in medical settings. Being harassed was the most prevalent discriminatory experience reported for financial- (73%), sex- (67%), race- (73%), and weight-based (75%) discrimination. In adjusted analyses, being harassed was attributed to financial- [OR = 0.61, 95% CI (0.55; 0.67), *p* < 0.001], race- [OR = 0.59, 95% CI (0.50; 0.69), *p* < 0.01] and weight discrimination [OR = 0.62, 95% CI (0.54; 0.71), *p* < 0.01] more frequently in the US than in England (Supplementary Table 4).

### Cross-national differences in perceived discrimination stratified by SEP

**Figure 2:**
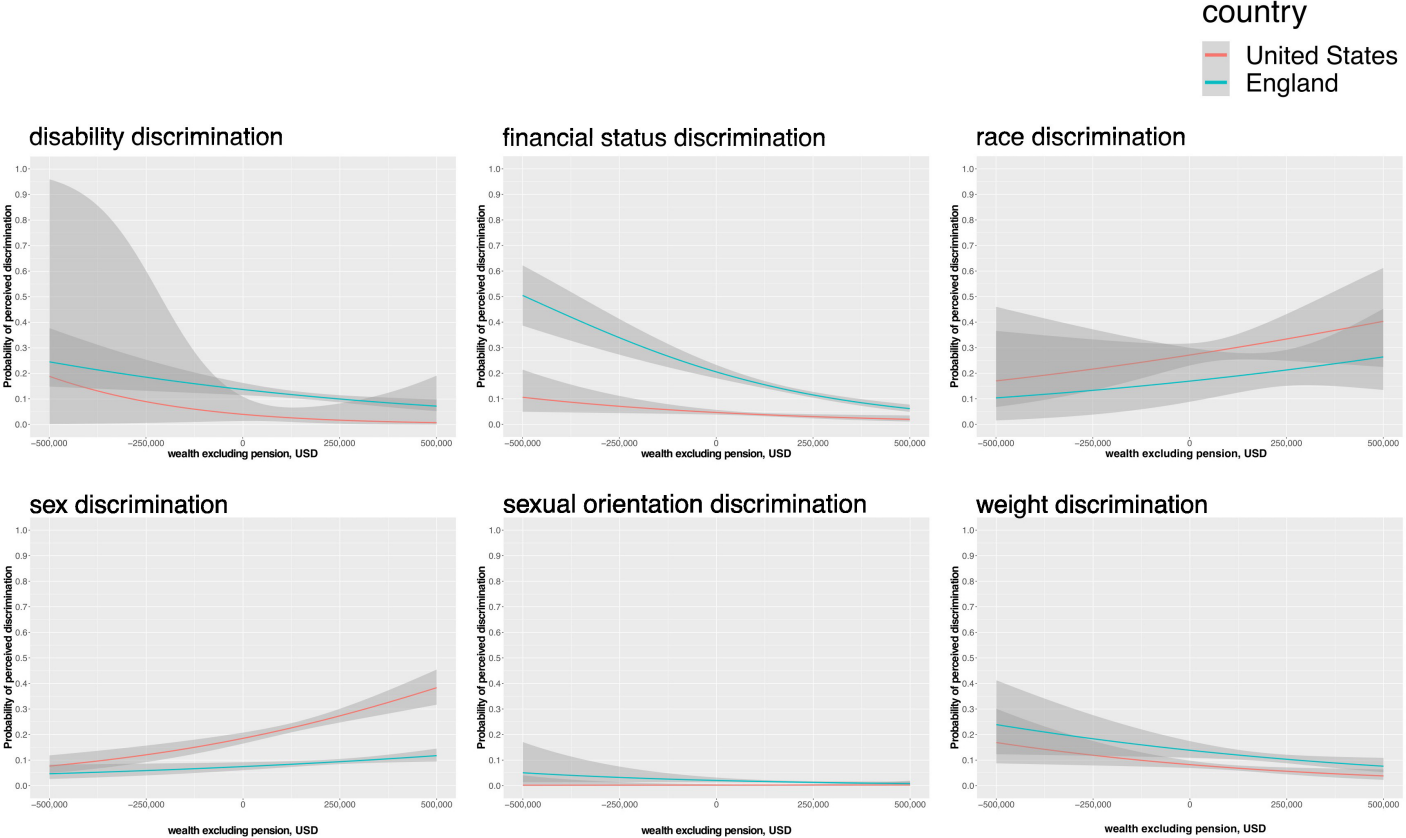
Probability of perceived discrimination (95%CI) predicted by wealth (USD) in England and US. Y-axis: the probability of perceived discrimination (yes, no); x-axis: wealth (USD)—a net sum of dept, financial wealth, housing, and physical wealth (i.e., land and jewelry). The probability of perceived discrimination predicted by wealth (USD) in England (green) and US (red) with 95% CI (in gray). Plots are produced from datasets restricted to the respective demographic or biometric characteristics [i.e., physical limitation, wealth > sample median (i.e., low socioeconomic position), ethnic minority, women, and BMI > 30]. Sexual orientation discrimination was not restricted to LGB. Wealth (USD). The analysis included ELSA (England) and HRS (US) cohorts.

Cross-national differences stratified by SEP are reported in Table 3. Figure 2 illustrates the probability of perceived discrimination as a function of wealth as estimated from unadjusted logistic regression models (95% CIs are plotted in gray). In adjusted analyses, for the low SEP groups, discrimination due to financial status remained significantly higher in England than in the US for the low SEP groups but the cross-national differences for disability and weight were no longer significant (Table 3).

**Table 3:** Prevalence and cross-national differences in perceived discrimination attributed to disability, financial status, sex, race, sexual orientation, and weight stratified by socioeconomic position (SEP).

**Attribution for**	**SEPS**	**Total** ***n***		**Perceived**				**Unadjusted cross-national**					**Adjusted cross-national**				
**discrimination**				**discrimination**, ***n*** **(%)**^**b**^				**difference**					**difference**				
		**England**	**US**	**England**		**US**		**OR^a^**	**95% CI**		**χ^2^**	***p*-value**	**OR**	**95% CI**		**χ^2^**	***p*-value**
Disability	Low SEP	2,662	332	259	9.73	6	1.81	3.36	1.47	7.66	8.58	**0.0034**	1.07	1.03	1.12	0.84	0.3587
	High SEP	195	54	3	1.54	1	1.85	0.39	0.04	3.94	0.00	0.9675	0.97	0.92	1.03	0.02	0.9006
Financial status	Low SEP	7,581	7,068	504	6.65	151	2.14	3.31	2.75	4.00	171.60	**0.0000**	1.08	1.07	1.10	13.04	**0.0003**
	High SEP	959	859	29	3.02	11	1.28	2.14	1.05	4.33	3.94	**0.0472**	1.03	1.00	1.06	0.15	0.6963
Sex (female)	Low SEP	4,257	4,131	374	8.79	512	12.39	0.34	0.30	0.40	219.16	**0.0000**	0.88	0.86	0.89	24.77	**0.0000**
	High SEP	486	452	57	11.73	57	12.61	0.37	0.24	0.55	23.04	**0.0000**	0.84	0.80	0.90	3.95	**0.0467**
Race (ethnic minority)	Low SEP	192	705	33	17.19	140	19.86	0.60	0.39	0.91	5.29	**0.0214**	0.93	0.85	1.01	0.55	0.4586
	High SEP	18	12	5	27.78	2	16.67	1.35	0.21	8.82	0.00	1.0000	1.31	0.89	1.93	0.34	0.5588
Sexual orientation*	NA	NA	NA	NA		NA		NA	NA	NA	NA	NA	NA	NA	NA	NA	NA
Weight (BMI > 30)	Low SEP	1,771	4,720	167	9.43	185	3.92	1.45	1.17	1.81	10.86	**0.0010**	1.04	1.02	1.05	1.20	0.2728
	High SEP	154	472	10	6.49	14	2.97	1.08	0.47	2.51	0.00	1.0000	1.00	0.95	1.05	0.00	0.9717

^a^ORs > 1 indicate the higher prevalence in England and ORs < 1 indicate the higher prevalence in the US. Values in bold meet the 0.05 *p*-value threshold.

^b^*n* (%) of individuals who perceived discrimination in low SEP and high SEP subgroups separately. BMI, Body Mass Index; CI, Confidence Interval; OR, Odds Ratio; SEP, socioeconomic position.

OR was adjusted for the following covariates: Disability: age and sex; Financial status: age and sex; Sex discrimination (female): age; Race: age and sex; Sexual orientation: no significant covariates; Weight: age, sex and wealth.

^*^Analysis was not stratified by SEP due to small *n*. Cross-national differences analysis stratified by the level of education is included in Supplementary Table 7.

Looking in England alone, there was a significant inverse wealth gradient in disability (ß = −0.61, *p* < 0.001), financial status (ß = −0.90, *p* < 0.001), and weight discrimination (ß = −0.46, *p* < 0.001), whereby discrimination was more likely to be reported by lower than higher SEP groups (Table 4; Figure 2). This wealth gradient was not significant in the US sample. Findings were mostly similar when wealth was modeled as a categorical variable (Supplementary Table 5).

**Table 4:** Wealth gradient in perceived discrimination: logistic regression coefficients describing the association between wealth (USD) and perceived discrimination.

	**Unadjusted**			**Adjusted**		
**Attributed reason**		**Standardized ß coefficient**	***z-*value**	***p*-value**	**Standardized ß coefficient**	***z-*value**	***p*-value**
Disability (physical limitation)	England	−0.61	−4.79	**0.0000**	−1.27	−4.81	**0.0000**
	US	−0.08	−0.27	0.7899	−0.25	−0.33	0.7430
Financial status	England	−0.90	−8.08	**0.0000**	−1.99	−8.41	**0.0000**
	US	−0.22	−1.00	0.3179	−0.03	−0.07	0.9428
Race (ethnic minority)	England	0.19	1.11	0.2655	0.39	1.08	0.2775
	US	−0.13	−0.61	0.5404	−0.25	−0.56	0.5723
Sex (female)	England	0.06	1.54	0.1242	0.03	0.81	0.4198
	US	0.10	2.81	**0.0049**	0.23	3.19	**0.0014**
Sexual orientation	England	−0.14	−0.92	0.3588	NA	NA	NA
	US	0.09	1.25	0.2107	NA	NA	NA
Weight (BMI > 30)	England	−0.46	−3.36	**0.0008**	−0.55	−2.31	**0.0210**
	US	−0.08	−1.05	0.2940	−0.01	−0.09	0.9256

Values in bold meet the 0.05 *p*-value threshold. BMI, Body Mass Index.

Wealth (USD)—a net sum of dept, financial wealth, housing, and physical wealth (i.e., land, and jewelry). ß-coefficient describing the association between between wealth and perceived discrimination in England and US. Analysis of the dataset restricted to the respective demographic or biometric characteristic [i.e., physical limitation, wealth > sample median (i.e., low socioeconomic position), ethnic minority, women, and BMI > 30]. Sexual orientation discrimination analysis was not restricted to lesbian, gay, bisexual individuals due to small *n*. Covariates: Disability: age and sex; Financial status: age and sex; Sex discrimination (female): age; Race: age and sex; Sexual orientation: no significant covariates; Weight: age and sex. The weighted logistic regression estimates are included in Supplementary Table 10.

Perceived race discrimination was more common in the US than in England in low SEP groups but not in the high SEP groups, in unadjusted analyses. However, in SEP-stratified analyses adjusted for sex, there was no longer a significant cross-country difference in perceived race discrimination. There was no significant wealth gradient for the probability of perceived race discrimination in either England or the US (Table 4; Figure 2).

Sex discrimination was more commonly reported in the US for both high and low SEP individuals. Looking in the US alone, there was a positive wealth gradient (ß = 0.10, *p* < 0.01) in the probability of perceived sex discrimination, suggesting higher SEP individuals were more likely to perceive discrimination (Table 4; Figure 2). This wealth gradient for sex discrimination was not evident in the English sample. In adjusted analyses stratified by SEP, cross-national differences in sex discrimination remained significant for both the low SEP group and high SEP group (Table 3). Sex discrimination was significantly more prevalent in the US than in England in low SEP group as well as high SEP group when adjusting for age.

When testing the interactive effects of SEP and country on the prevalence of perceived discrimination attributed to each characteristic separately, there was a statistically significant interaction effect between SEP and country on the prevalence of perceived financial status discrimination among the overall sample (ß = −0.05, SE = 0.02, *p* < 0.01) but no other discrimination types (Supplementary Table 6).

### Results of the sensitivity analysis

The cross-national comparison stratified by SEP operationalized using education level did not differ from the main results (Supplementary Table 7). When education was included as a covariate, the differences in perceived sex discrimination were attenuated to non-significant (Supplementary Tables 8, 9). No other differences between this sensitivity analysis and the main findings were observed.

The weighted estimates, accounting for the selection bias in ELSA and HRS separately did not deviate from the main results, when assessing the association between wealth and perceived discrimination (Supplementary Table 10).

## Discussion

This study examined differences in perceived discrimination across multiple characteristics experienced by older and middle-aged adults living in England and the US. Financial status discrimination was more common in England than in the US, affecting individuals of low SEP. Sexual orientation discrimination was also perceived more frequently in England than in the US. More women perceived sex discrimination in the US than in England, in both high and low SEP groups. In the US, but not in the English sample, there was a positive wealth gradient in perceived sex discrimination. When comparing low SEP groups in unadjusted analyses, financial status, disability, and weight discrimination were more prevalent in England than in the US. Looking at England alone, we identified an inverse wealth gradient in disability-, financial status- and perceived weight discrimination in England, in both unadjusted and adjusted analyses. This was not found in the US sample. Perceived race discrimination was more prevalent in the US but only in analyses where there had been no adjustment for weight, age, sex, and wealth.

### Discrimination types that are more common in England

We found financial status discrimination to be more widespread in England than in the US, independent of age, sex, and wealth. Our stratified analyses suggest this result is driven by low SEP groups. This finding aligns with earlier cross-national research in these cohorts, which found higher rates of perceived age discrimination in low SEP groups in England than in the US (22). Wealth inequality is high in both England (31) and the US (26). The history of a hierarchically organized society in England (16) and the common belief that the US is more economically egalitarian (17) may partially explain the difference.

A growing literature suggests that LGB adults have poorer health and wellbeing than their heterosexual counterparts (32). We investigated rates of sexual orientation discrimination in lesbian, gay, and bisexual (LGB) adults and observed higher reports of sexual orientation discrimination in England than in the US. We were unable to conduct adjusted analyses due to small numbers identifying as LGB, so caution is needed in interpreting our findings. However, our findings add to the limited research on middle-aged and older LGB populations (14). Longitudinal work in ELSA indicates that perceived discrimination is associated with poorer wellbeing in LGB participants, particularly when the discrimination is attributed to sexual orientation (14). This suggests the age-related burden of poor health and wellbeing in LGB groups may be compounded by discrimination, especially in England. Older adults in England grew up at a time when homosexuality was classified as a mental illness and sex between two men was illegal (33, 34). Middle-aged adults grew up at a time when schools were banned from teaching about homosexuality (35) and there was the onset of the AIDS epidemic. These and other negative experiences may have had lasting effects on participants' perceptions of discrimination. However, the social context was also challenging in the US and the cross-national difference needs further research.

In unadjusted analyses, the prevalence of disability discrimination was higher in England than in the US. The English sample included more individuals living with disability compared to the US sample. This is in keeping with international estimates showing that a slightly greater proportion of the population of the UK is in receipt of disability payments than in the US (36). Similarly, there were more individuals who perceived disability discrimination in England than in the US. This finding was patterned by SEP, as the association for high SEP groups was attenuated to the null in stratified analyses. In the UK, approximately one in five people self-report disability (37) and 4 million older adults (36% of people aged 65–74, and 47% of those aged 75+) live with a limiting long-standing illness (38). The practical difficulties associated with disability may be aggravated by discrimination. Previous work in ELSA (11, 12) and other UK cohorts (6) suggests that perceived discrimination has compounding adverse effects on wellbeing in those with disability. Further, in England, reports of perceived discrimination were inversely related to wealth. Therefore, perceived disability discrimination, if unaddressed, could place an additional burden on marginalized aging English adults.

In unadjusted analyses, individuals with obesity perceived weight discrimination more frequently in England than in the US. This difference was socially patterned, as the association remained for low SEP groups alone in stratified analyses. There was no significant difference after adjustment for age and sex. Obesity is common in both countries, particularly in low SEP groups, though rates are consistently higher in the US (39, 40). The “normalization” of obesity may have resulted in reduced perceived discrimination based on this attribute in the US (27). In ELSA, weight discrimination has been prospectively linked with poorer wellbeing (13), and cross-national comparisons suggest that the impact of obesity on wellbeing is partially mediated by discrimination (9, 41). Weight-based perceived discrimination is associated with poor health behaviors (42) which may further harm health, particularly for marginalized groups, as there is a recognized wealth gradient in health behaviors (43).

### Discrimination types that are more common in the US

Women in the US perceived sex discrimination more frequently than women in England, independent of age and wealth. This discrepancy may mirror differences in gender inequality between the countries, as in 2010 and 2020, the US ranked twice as high on the global gender inequality index as the UK (44), based on economic, educational, and political disparities. Further research is needed to evaluate the impact of political under-representation and economic and employment disempowerment on reports of sex discrimination in the population. We observed a wealth gradient in sex discrimination in the US. This is in keeping with earlier work in younger samples (7). It is unclear why high SEP women report more encounters with sexism. One possibility is that they may recognize sexism more readily, for example by being able to distinguish it from wealth-based discrimination. More research is needed to investigate this possibility.

In unadjusted comparisons, perceived racial discrimination was more common in the US than in England. This difference did not remain in adjusted analyses. Caution is needed in interpreting these findings due to the low proportion of ethnic minorities in both samples (25, 26). However, this was the most reported form of perceived discrimination in both English and US cohorts. This is concerning considering the increased recognition of the impact of racial discrimination on mental health (45, 46) as well as emerging evidence on physical health impacts (5, 47).

### Strengths and limitations

This study contributes to the evidence concerning the prevalence of distinct types of perceived discrimination in England and the US, using nationally representative samples of adults aged 50 and over. We have employed a harmonized measure perceived discrimination cross-nationally. In addition, cross-national differences were assessed stratifying by SEP, which may have improved precision and also elucidated a significant wealth gradient in perceived discrimination in middle-aged and older adults. However, the study is not without limitations. The analyzed sample had few ethnic minorities and LGB participants, reducing the generalizability of the findings. Unweighted estimated for the cross-national comparisons were used because ELSA and HRS are weighted differently. Study results reflect self-reported perceptions of discrimination rather than objective encounters with discrimination. In addition, as in other observational studies, the findings may be prone to the bias introduced by unmeasured confounding. Finally, future research is encouraged to include additional types of perceived discrimination (e.g., attributed to transgender and gender non-conforming status).

## Conclusion

The cross-national differences in perceived discrimination identified in the study can inform country-specific policies and interventions targeted at middle-aged and older adults, with the ultimate aim of alleviating the negative impact of perceived discrimination on health and wellbeing. This study highlights the importance of considering contextual moderators such as SEP and sociocultural context in discrimination research.

## Data availability statement

Publicly available datasets were analyzed in this study. This data can be found here: https://g2aging.org/downloads.

## Ethics statement

The studies involving human participants were reviewed and approved by ELSA was approved by the London Multicentre Research and Ethics Committee (MREC/01/02/91). Approval for HRS was obtained from the University of Michigan Institutional Review Board (https://hrs.isr.umich.edu/publications/biblio/9048). The patients/participants provided their written informed consent to participate in this study.

## Author contributions

RH secured funding for this study and conceived the study. RH and AA contributed to study design. AA carried out the statistical analysis and drafted the manuscript. KR and RH provided critical comments and revisions to the manuscript. All authors contributed to the article and approved the submitted version.

## Funding

This project was supported by the Academy of Medical Sciences/the Wellcome Trust/the Government Department of Business, Energy and Industrial Strategy/the British Heart Foundation/Diabetes UK Springboard Award (SBF006\1036).

## Conflict of interest

The authors declare that the research was conducted in the absence of any commercial or financial relationships that could be construed as a potential conflict of interest. The reviewer AR declared a shared affiliation with all of the authors, to the handling editor at time of review.

## Publisher's note

All claims expressed in this article are solely those of the authors and do not necessarily represent those of their affiliated organizations, or those of the publisher, the editors and the reviewers. Any product that may be evaluated in this article, or claim that may be made by its manufacturer, is not guaranteed or endorsed by the publisher.
