# Supplementary material for: Perceived discrimination in middle-aged and older adults: Comparison between England and the United States
Source: Front Public Health. 2022 Nov 10;10:975776. doi: 10.3389/fpubh.2022.975776 (PMC9685535; doi:10.3389/fpubh.2022.975776)
Supplement: Supplementary file 1 [file Data_Sheet_1.docx]

**Supplementary Table 1. Unadjusted OR and Adjusted OR for cross-national differences in ageism between US and UK (US = 0; England = 1)**

|  | **Total N** | | **Perceived discrimination, n (%)** | | | | ***OR*** | ***95% CI*** | | ***χ2*** | ***p-value*** |
| --- | --- | --- | --- | --- | --- | --- | --- | --- | --- | --- | --- |
|  | ***England*** | ***USA*** | ***England*** | | ***USA*** | |  |  |  |  |  |
| **Unadjusted** | 8671 | 7927 | 1328 | 15.32 | 1331 | 16.79 | 2.34 | 2.08 | 2.65 | 192.99 | **0.000** |
| **Adjusted: age, sex, wealth** |  |  | ` |  |  |  | 1.18 | 1.15 | 1.21 | 29.48 | **0.000** |

**Note.** ^1^*OR*s greater than 1 indicate higher prevalence in England and ORs less than 1 indicate the higher prevalence in the US. **CI**= Confidence Interval; **OR** – Odds Ratio; **SES** – socioeconomic status.

**Supplementary Table 2. Cross-national differences in ageism between US and UK (US = 0; England = 1) stratified by SES.**

|  | **SES level** | **Total N** | | **Perceived discrimination** | | **Unadjusted** | | | | | **Adjusted** | | | | |
| --- | --- | --- | --- | --- | --- | --- | --- | --- | --- | --- | --- | --- | --- | --- | --- |
|  |  | ***ELSA*** | ***HRS*** | ***ELSA*** | ***HRS*** | ***OR*** | ***95% CI*** | | ***χ2*** | ***p-value*** | ***OR*** | ***95% CI*** | | ***χ2*** | ***p-value*** |
| **Age discrimination** | Low SES | 7581 | 7068 | 1186 | 1179 | 2.51 | 2.21 | 2.86 | 201.10 | **0.000** | 1.19 | 1.16 | 1.23 | 30.65 | **0.000** |
|  | High SES | 959 | 859 | 130 | 152 | 1.22 | 0.84 | 1.79 | 0.92 | 0.3386 | 1.08 | 0.99 | 1.17 | 0.61 | 0.4357 |

**Note.** ^1^*OR*s greater than 1 indicate higher prevalence in England and ORs less than 1 indicate the higher prevalence in the US. **CI**= Confidence Interval; **OR** – Odds Ratio; **SES** – socioeconomic status.

**Supplementary Table 3. Cross-national differences in the frequency of situations in which discrimination was experienced.**

| **Attributed reason** | **Situation** | **Country** | **Perceived discrimination** | | **Unadjusted cross-national difference** | | | | | **Adjusted cross-national difference** | | | | | | |
| --- | --- | --- | --- | --- | --- | --- | --- | --- | --- | --- | --- | --- | --- | --- | --- | --- |
|  |  |  | ***n*** | ***%*** | ***OR^1^*** | ***95% CI*** | | ***χ2*** | ***p-value*** | ***OR^1^*** | ***95% CI*** | | ***χ2*** | | ***p-value*** | |
| **Disability** | **Less respect** | ***USA*** | 3 | 42.86 |  |  |  |  |  |  |  |  | |  | |  |
|  |  | ***UK*** | 205 | 77.65 | 4.88 | 1.06 | 22.45 | 3.15 | **0.0757** | 1.31 | 0.94 | 1.81 | | 0.42 | | 0.5178 |
|  | **Medical** | ***USA*** | 6 | 85.71 |  |  |  |  |  |  |  |  | |  | |  |
|  |  | ***UK*** | 56 | 21.21 | 0.05 | 0.01 | 0.38 | 12.56 | **0.0004** | 0.57 | 0.40 | 0.79 | | 1.91 | | 0.1667 |
|  | **Not clever** | ***USA*** | 4 | 57.14 |  |  |  |  |  |  |  |  | |  | |  |
|  |  | ***UK*** | 167 | 63.26 | 1.39 | 0.30 | 6.35 | 0.00 | 0.9782 | 1.06 | 0.72 | 1.58 | | 0.02 | | 0.8797 |
|  | **Poor service** | ***USA*** | 3 | 42.86 |  |  |  |  |  |  |  |  | |  | |  |
|  |  | ***UK*** | 100 | 37.88 | 0.87 | 0.19 | 3.98 | 0.00 | 1.0000 | 0.91 | 0.61 | 1.35 | | 0.05 | | 0.8172 |
|  | **Harassed** | ***USA*** | 6 | 85.71 |  |  |  |  |  |  |  |  | |  | |  |
|  |  | ***UK*** | 77 | 29.17 | 0.07 | 0.01 | 0.60 | 7.46 | **0.0063** | 0.53 | 0.37 | 0.75 | | 2.44 | | 0.1180 |
| **Financial discrimination** | **Less respect** | ***USA*** | 41 | 27.15 |  |  |  |  |  |  |  |  | |  | |  |
|  |  | ***UK*** | 322 | 63.89 | 4.80 | 3.21 | 7.18 | 62.85 | **0.0000** | 1.45 | 1.30 | 1.63 | | 8.99 | | **0.0027** |
|  | **Medical** | ***USA*** | 106 | 70.20 |  |  |  |  |  |  |  |  | |  | |  |
|  |  | ***UK*** | 57 | 11.31 | 0.05 | 0.03 | 0.08 | 214.50 | **0.0000** | 0.56 | 0.51 | 0.61 | | 21.85 | | **0.0000** |
|  | **Not clever** | ***USA*** | 65 | 43.05 |  |  |  |  |  |  |  |  | |  | |  |
|  |  | ***UK*** | 224 | 44.44 | 1.06 | 0.73 | 1.53 | 0.05 | 0.8232 | 1.00 | 0.88 | 1.13 | | 0.00 | | 0.9918 |
|  | **Poor service** | ***USA*** | 71 | 47.02 |  |  |  |  |  |  |  |  | |  | |  |
|  |  | ***UK*** | 185 | 36.71 | 0.64 | 0.44 | 0.93 | 5.09 | **0.0240** | 0.89 | 0.79 | 1.00 | | 0.87 | | 0.3514 |
|  | **Harassed** | ***USA*** | 109 | 72.19 |  |  |  |  |  |  |  |  | |  | |  |
|  |  | ***UK*** | 93 | 18.45 | 0.08 | 0.05 | 0.12 | 160.01 | **0.0000** | 0.61 | 0.55 | 0.67 | | 15.77 | | **0.0001** |
| **Sex discrimination, female** | **Less respect** | ***USA*** | 98 | 17.22 |  |  |  |  |  |  |  |  | |  | |  |
|  |  | ***UK*** | 348 | 79.63 | 18.93 | 13.75 | 26.05 | 387.77 | **0.0000** | 1.87 | 1.78 | 1.96 | | 95.12 | | **0.0000** |
|  | **Medical** | ***USA*** | 379 | 66.61 |  |  |  |  |  |  |  |  | |  | |  |
|  |  | ***UK*** | 50 | 11.44 | 0.06 | 0.05 | 0.09 | 303.98 | **0.0000** | 0.58 | 0.55 | 0.61 | | 74.05 | | **0.0000** |
|  | **Not clever** | ***USA*** | 117 | 20.56 |  |  |  |  |  |  |  |  | |  | |  |
|  |  | ***UK*** | 229 | 52.40 | 4.26 | 3.23 | 5.62 | 109.37 | **0.0000** | 1.38 | 1.30 | 1.46 | | 24.84 | | **0.0000** |
|  | **Poor service** | ***USA*** | 210 | 36.91 |  |  |  |  |  |  |  |  | |  | |  |
|  |  | ***UK*** | 176 | 40.27 | 1.15 | 0.89 | 1.49 | 1.05 | 0.3060 | 1.04 | 0.97 | 1.10 | | 0.30 | | 0.5849 |
|  | **Harassed** | ***USA*** | 381 | 66.96 |  |  |  |  |  |  |  |  | |  | |  |
|  |  | ***UK*** | 86 | 19.68 | 0.12 | 0.09 | 0.16 | 222.24 | **0.0000** | 0.62 | 0.59 | 0.66 | | 54.46 | | **0.0000** |
| **Race discrimination** | **Less respect** | ***USA*** | 33 | 23.24 |  |  |  |  |  |  |  |  | |  | |  |
|  |  | ***UK*** | 25 | 65.79 | 6.88 | 3.12 | 15.18 | 24.35 | **0.0000** | 1.57 | 1.34 | 1.84 | | 5.82 | | **0.0159** |
|  | **Medical** | ***USA*** | 93 | 65.49 |  |  |  |  |  |  |  |  | |  | |  |
|  |  | ***UK*** | 7 | 18.42 | 0.12 | 0.05 | 0.30 | 23.97 | **0.0000** | 0.65 | 0.55 | 0.76 | | 5.51 | | **0.0189** |
|  | **Not clever** | ***USA*** | 46 | 32.39 |  |  |  |  |  |  |  |  | |  | |  |
|  |  | ***UK*** | 24 | 63.16 | 3.77 | 1.76 | 8.08 | 11.24 | **0.0008** | 1.39 | 1.17 | 1.65 | | 3.10 | | 0.0782 |
|  | **Poor service** | ***USA*** | 47 | 33.10 |  |  |  |  |  |  |  |  | |  | |  |
|  |  | ***UK*** | 15 | 39.47 | 1.44 | 0.68 | 3.05 | 0.59 | 0.4425 | 1.10 | 0.92 | 1.31 | | 0.27 | | 0.6051 |
|  | **Harassed** | ***USA*** | 103 | 72.54 |  |  |  |  |  |  |  |  | |  | |  |
|  |  | ***UK*** | 8 | 21.05 | 0.10 | 0.04 | 0.24 | 31.47 | **0.0000** | 0.59 | 0.50 | 0.69 | | 8.15 | | **0.0043** |
| **Sexual orientation discrimination** | **Less respect** | ***USA*** | 4 | 33.33 |  |  |  |  |  | NA | NA | NA | | NA | | NA |
|  |  | ***UK*** | 35 | 56.45 | 2.59 | 0.71 | 9.52 | 1.33 | 0.2492 |  |  |  | |  | |  |
|  | **Medical** | ***USA*** | 9 | 75.00 |  |  |  |  |  |  |  |  | |  | |  |
|  |  | ***UK*** | 12 | 19.35 | 0.08 | 0.02 | 0.35 | 12.40 | **0.0004** |  |  |  | |  | |  |
|  | **Not clever** | ***USA*** | 5 | 41.67 |  |  |  |  |  |  |  |  | |  | |  |
|  |  | ***UK*** | 21 | 33.87 | 0.72 | 0.20 | 2.53 | 0.04 | 0.8513 |  |  |  | |  | |  |
|  | **Poor service** | ***USA*** | 5 | 41.67 |  |  |  |  |  |  |  |  | |  | |  |
|  |  | ***UK*** | 21 | 33.87 | 0.75 | 0.21 | 2.67 | 0.01 | 0.9126 |  |  |  | |  | |  |
|  | **Harassed** | ***USA*** | 7 | 58.33 |  |  |  |  |  |  |  |  | |  | |  |
|  |  | ***UK*** | 9 | 14.52 | 0.12 | 0.03 | 0.48 | 8.73 | **0.0031** |  |  |  | |  | |  |
| **Weight discrimination, BMI>30** | **Less respect** | ***USA*** | 39 | 19.60 |  |  |  |  |  |  |  |  | |  | |  |
|  |  | ***UK*** | 147 | 82.58 | 19.85 | 11.73 | 33.60 | 146.70 | **0.0000** | 1.74 | 1.54 | 1.97 | | 13.18 | | **0.0003** |
|  | **Medical** | ***USA*** | 145 | 72.86 |  |  |  |  |  |  |  |  | |  | |  |
|  |  | ***UK*** | 28 | 15.73 | 0.07 | 0.04 | 0.12 | 121.23 | **0.0000** | 0.56 | 0.50 | 0.64 | | 14.08 | | **0.0002** |
|  | **Not clever** | ***USA*** | 68 | 34.17 |  |  |  |  |  |  |  |  | |  | |  |
|  |  | ***UK*** | 97 | 54.49 | 2.36 | 1.55 | 3.59 | 15.58 | **0.0001** | 1.36 | 1.18 | 1.57 | | 3.98 | | **0.0459** |
|  | **Poor service** | ***USA*** | 100 | 50.25 |  |  |  |  |  |  |  |  | |  | |  |
|  |  | ***UK*** | 72 | 40.45 | 0.69 | 0.46 | 1.04 | 2.75 | 0.0975 | 0.92 | 0.79 | 1.07 | | 0.28 | | 0.5956 |
|  | **Harassed** | ***USA*** | 150 | 75.38 |  |  |  |  |  |  |  |  | |  | |  |
|  |  | ***UK*** | 38 | 21.35 | 0.08 | 0.05 | 0.14 | 110.28 | **0.0000** | 0.62 | 0.54 | 0.71 | | 9.52 | | **0.0020** |

*Note.* ***χ2 -*** Pearson's chi-squared test**; CI=** Confidence Interval; **BMI** – body Mass Index**; OR** – Odds Ratio; **SES** – socioeconomic status.

^1^ *OR*s greater than 1 indicate the higher prevalence in England and *OR*s less than 1 indicate the higher prevalence in the USA. *OR* was adjusted for the following covariates: *Disability*: age; *Financial status*: age, sex, and wealth; *Sex discrimination (female):* age and wealth; Race: sex; *Sexual orientation*: no significant covariates; *Weight*: age, sex and wealth.

**Supplementary Table 4. Within-country differences in the prevalence of perceived discrimination attributed to disability, financial status, sex, race, sexual orientation, and weight between low and high SES.**

| **Attribution for discrimination** | **Country** | **Total N** | | **Perceived discrimination, N (%)** | | | | | **Unadjusted diff. between low and high SES** | | | | | | | | **Adjusted diff. between low and high SES** | | | | |
| --- | --- | --- | --- | --- | --- | --- | --- | --- | --- | --- | --- | --- | --- | --- | --- | --- | --- | --- | --- | --- | --- |
|  |  | ***Low SES*** | ***High SES*** | ***Low SES*** | | | ***High SES*** | | ***OR*** | | ***95% CI*** | | | ***χ2*** | | ***p-value*** | ***OR*** | ***95% CI*** | | ***χ2*** | ***p-value*** |
| **Disability**  **(physical limitation)** | ***England*** | 2662 | 195 | 259 | 9.73 | 3 | | 1.54 | 0.14 | 0.05 | | 0.45 | 13.90 | | **0.0002** | | 0.92 | 0.86 | 0.97 | 0.67 | 0.4115 |
| **Financial status** | ***England*** | 7581 | 959 | 504 | 6.65 | 29 | | 3.02 | 0.46 | 0.31 | | 0.67 | 15.86 | | **0.0001** | | 0.96 | 0.90 | 1.02 | 0.17 | 0.6828 |
| **Sex (female)** | ***England*** | 4257 | 486 | 374 | 8.79 | 57 | | 11.73 | 1.34 | 1.00 | | 1.80 | 3.47 | | 0.0623 | | 1.02 | 0.98 | 1.06 | 0.08 | 0.7774 |
| **Race**  **(ethnic minority)** | ***England*** | 192 | 18 | 33 | 17.19 | 5 | | 27.78 | 1.68 | 0.56 | | 5.03 | 0.38 | | 0.5354 | | 1.09 | 0.90 | 1.32 | 0.14 | 0.7129 |
| **Sexual orientation*** | ***England*** | 7581 | 959 | 58 | 0.77 | 4 | | 0.42 | 0.58 | 0.21 | | 1.61 | 0.71 | | 0.4000 | | NA | NA | NA | NA | NA |
| **Weight (BMI>30)** | ***England*** | 1771 | 154 | 167 | 9.43 | 10 | | 6.49 | 0.67 | 0.35 | | 1.30 | 1.10 | | 0.2951 | | 1.03 | 0.95 | 1.12 | 0.04 | 0.8510 |
| **Disability (physical limitation)** | ***USA*** | 332 | 54 | 6 | 1.81 | 1 | | 1.85 | 1.22 | 0.14 | | 10.59 | 0.00 | | 1.0000 | | 1.00 | 0.93 | 1.08 | 0.00 | 0.9873 |
| **Financial status** | ***USA*** | 7068 | 859 | 151 | 2.14 | 11 | | 1.28 | 0.71 | 0.38 | | 1.32 | 0.90 | | 0.3441 | | 0.99 | 0.96 | 1.01 | 0.03 | 0.8583 |
| **Sex (female)** | ***USA*** | 4131 | 452 | 512 | 12.39 | 57 | | 12.61 | 1.26 | 0.92 | | 1.74 | 1.81 | | 0.1784 | | 0.96 | 0.88 | 1.04 | 0.18 | 0.6716 |
| **Race (ethnic minority)** | ***USA*** | 705 | 12 | 140 | 19.86 | 2 | | 16.67 | 0.74 | 0.15 | | 3.62 | 0.00 | | 1.0000 | | 0.94 | 0.70 | 1.26 | 0.04 | 0.8440 |
| **Sexual orientation*** | ***USA*** | 7068 | 859 | 9 | 0.13 | 3 | | 0.35 | 3.31 | 0.89 | | 12.26 | 1.95 | | 0.1630 | | NA | NA | NA | NA | NA |
| **Weight (BMI>30)** | ***USA*** | 4720 | 472 | 185 | 3.92 | 14 | | 2.97 | 0.90 | 0.51 | | 1.58 | 0.05 | | 0.8147 | | 1.01 | 0.97 | 1.06 | 0.02 | 0.8951 |

*Note.* ***χ2 -*** Pearson's chi-squared test**; CI=** Confidence Interval; **BMI** – body Mass Index**; OR** – Odds Ratio; **SES** – socioeconomic status.

***** Unrestricted analysis due to small n. 1. Low SES was coded as 0 and high SES as 1.

ORs greater than 1 indicate the higher prevalence in high SES and ORs less than 1 indicate the higher prevalence in low SES. OR were adjusted for the following covariates: *Disability*: age; *Financial status*: age, and sex; *Sex discrimination (female):* age; Race: sex; *Sexual orientation*: no significant covariates; *Weight*: age, and sex.

**Supplementary Table 5. The interaction effect on perceived discrimination between SES and country.**

| **Attributed reason** | ***ß*-coefficient** | **SE** | ***t-value*** | ***p-value*** |
| --- | --- | --- | --- | --- |
| Disability (physical limitation) | -0.09 | 0.07 | -1.43 | 0.1516 |
| Financial status | -0.05 | 0.02 | -2.64 | **0.0083** |
| Sex (female) | -0.02 | 0.03 | -0.53 | 0.5959 |
| Race (ethnic minority) | 0.15 | 0.18 | 0.81 | 0.4187 |
| Sexual orientation | -0.01 | 0.01 | -1.71 | 0.0869 |
| Weight (BMI>30) | -0.02 | 0.03 | -0.79 | 0.4317 |

Note. **BMI** – Body Mass Index. Negative ß-coefficient indicates more prevalent discrimination in lower SES in England than in low SES US and high SES in both countries.

**Supplementary Table 6. Wealth gradient in perceived discrimination: logistic regression coefficients describing the association between wealth (USD) and perceived discrimination (weighted to account for selection bias).**

|  |  | **Unadjusted** |  | **Adjusted** |  |
| --- | --- | --- | --- | --- | --- |
| **Attributed reason** |  | Standardized  ß coefficient | *p*-value | Standardized  ß coefficient | *p*-value |
| Disability (physical limitation) | England | -0.41 | **0.0000** | -1.16 | **0.0000** |
|  | US | -0.04 | 0.8799 | -0.14 | 0.7560 |
| Financial status | England | -0.86 | **0.0000** | -1.98 | **0.0000** |
|  | US | -0.18 | 0.2171 | -0.01 | 0.9657 |
| Race (ethnic minority) | England | 0.23 | 0.3655 | 0.39 | 0.2740 |
|  | US | -0.12 | 0.6404 | -0.23 | 0.5892 |
| Sex (female) | England | 0.38 | 0.1242 | 0.02 | 0.5133 |
|  | US | 0.19 | **0.0069** | 0.21 | **0.0027** |
| Sexual orientation | England | -0.12 | 0.4588 | NA | NA |
|  | US | 0.41 | 0.2407 | NA | NA |
| Weight (BMI>30) | England | -0.52 | **0.0008** | -0.41 | **0.0280** |
|  | US | -0.09 | 0.1940 | -0.02 | 0.9370 |

**Note.** **BMI** – Body Mass Index. Wealth (USD) – a net sum of dept, financial wealth, housing, and physical wealth (i.e., land, businesses, and jewelry). ß-coefficient describing the association between wealth perceived discrimination in England and US. Analysis of the dataset restricted to the respective demographic or biometric characteristic (i.e., physical limitation, wealth>sample median (i.e., low SES), ethnic minority, women, and BMI>30). Sexual orientation discrimination analysis was not restricted to lesbian, gay, bisexual individuals due to small *n*. Covariates: *Disability*: age and sex; *Financial status*: age and sex; *Sex discrimination (female):* age; Race: age and sex; *Sexual orientation*: no significant covariates; *Weight*: age and sex.
